# A Neutral Triple-Helix Pomegranate (*Punica granatum*) Peel Polysaccharide with Lipid-Lowering and Prebiotic Activities

**DOI:** 10.1021/acs.jafc.5c16652

**Published:** 2026-04-28

**Authors:** Hui-Xian Shen, Le Shi, Yan-Xia Li, Xiao-Yu Zhang, Chun-Qiong Zhao, Hang Ma, Yong-Ming Lu

**Affiliations:** † School of Life Sciences and Medical Engineering, 12487 Anhui University, Hefei, Anhui 230601, P. R. China; ‡ Bioactive Botanical Research Laboratory, Department of Biomedical and Pharmaceutical Sciences, College of Pharmacy, University of Rhode Island, Kingston, Rhode Island 02881, United States

**Keywords:** pomegranate peel polysaccharide, lipid metabolism, HepG2 cells, 16S rRNA, untargeted metabolomics

## Abstract

A homogeneous neutral polysaccharide (PPP-1; 1.5 × 10^4^ Da) was isolated from pomegranate peel and characterized as a glucose-rich glucan with α-/β-pyranose linkages and a triple-helix conformation. PPP-1 improved lipid dysregulation in oleic acid-treated HepG2 cells, reducing total cholesterol and triglycerides (TG) by up to 76.2 and 70.4%, respectively, and lowering low-density lipoprotein cholesterol (LDL-C) while increasing high-density lipoprotein cholesterol (HDL-C), accompanied by suppression of SREBP-1c and FAS via SIRT1/AMPK activation. In high-fat diet (HFD)-fed mice, PPP-1 reduced weight gain and epididymal fat by 42.1 and 41.5%, alleviated hepatic steatosis, and lowered alanine aminotransferase/aspartate aminotransferase by 27.9 and 32.1% without affecting food intake. PPP-1 also corrected HFD-induced dyslipidemia, increasing HDL-C by 36.4% and reducing TG and LDL-C by 37.0% and 35.4%. PPP-1 lowered the *Firmicute*s/*Bacteroidetes* ratio and enriched *Lactobacillus* and *Bifidobacterium*. Metabolomics confirmed normalization of obesity-related metabolites and modulation of lipid-related pathways. These findings identify PPP-1 as a dual-acting hepatic and prebiotic polysaccharide with promise for metabolic health.

## Graphical abstract

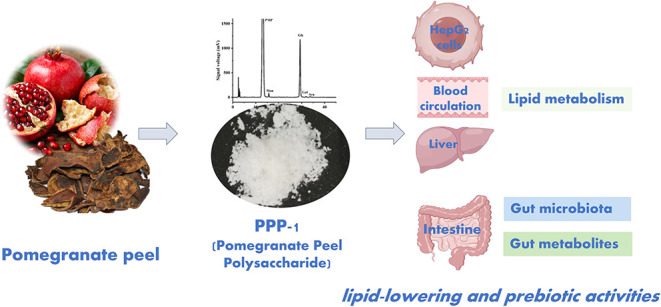

## 1 Introduction

Lipids are essential biomolecules that play pivotal roles in cell membrane integrity, energy storage, hormone synthesis, and immune regulation.^1^ However, chronic overconsumption of high-fat diets has led to a global surge in metabolic disorders such as obesity, metabolic-associated fatty liver disease, and cardiovascular disease.^2^ These disorders are driven by systemic lipid dysregulation, insulin resistance, and excessive ectopic fat accumulation in nonadipose tissues, processes that trigger oxidative stress and low-grade inflammation.^3,4^ Although pharmacological therapies are available, their long-term use often entails side effects, underscoring the urgent need for safe nutritional strategies to restore lipid homeostasis. This has fueled growing interest in the discovery of interventions with bioactive compounds from natural sources.^5^

Among these bioactivities, polysaccharides have emerged as promising modulators of lipid metabolism due to their structural diversity, favorable safety profile, and prebiotic potential. Dietary polysaccharides from various sources, such as tea, fungi, and edible plants, have demonstrated hypolipidemic effects through multitarget mechanisms involving bile acid metabolism, gut microbiota remodeling, and suppression of inflammatory signaling.^6^ For instance, polysaccharides from large-leaf yellow tea attenuated hepatic cholesterol accumulation and reshaped gut microbiota through the FXR-SHP signaling pathway and bile salt hydrolase pathways.^7^ Similarly, polysaccharides from *Sanghuangporus vaninii* reduced adiposity and steatosis via modulation of the TLR4/NF-κB axis.^8^ These findings highlight the potential of natural polysaccharides in the regulation of lipid metabolism via multitarget mechanisms.

In parallel, valorization of agricultural byproducts offers a sustainable approach to discovering such bioactivities. Pomegranate (*Punica granatum* L.) peel accounts for nearly half of the fruit’s weight and represents the major residue of the juice industry.^9^ While extensively studied for its phenolic antioxidants and ellagitannins, its polysaccharide fraction remains relatively underexplored.^10,11^ Previous reports have studied pomegranate peel polysaccharides (PPPs) for their immunostimulatory,^12^ antioxidant,^13^ anticancer^14^ and gelation properties.^15^ The majority of reported PPPs are acidic, pectic-type heteropolysaccharides rich in galacturonic acid; however, their homogeneity, higher-order conformation, and structure–function relationships have not been fully elucidated. Furthermore, the discovery of novel polysaccharides from pomegranate peel and the characterization of their specific metabolic regulatory roles remains limited.

Recent evidence suggests that the chemical architecture of polysaccharides, *i*.*e*., monosaccharide composition, glycosidic linkage, molecular weight, and conformation, strongly influences their biological activity.^16^ β-Glucans, for example, are known to activate AMPK signaling and promote lipid catabolism, while triple-helical configurations enhance receptor recognition and biological stability.^17^ These insights prompted us to revisit the pomegranate peel to (1) isolate and characterize structurally defined polysaccharides, and (2) correlate their chemistry to lipid metabolism regulatory effects. Therefore, this study aimed to isolate and chemically characterize a homogeneous PPP (using comprehensive spectroscopic methods) and elucidate its mechanism in lipid metabolic regulation (using both *in vitro* steatosis models and an *in vivo* high-fat diet-induced obese mouse model).

## 2 Materials and Methods

### 2.1 Materials and Chemicals

Pomegranates were purchased from a local market (Huaibei, Anhui, China) and a voucher specimen (No. 202201003) has been deposited in Anhui University Herbarium. Trifluoroacetic acid and concentrated sulfuric acid were purchased from National Pharmaceutical Group Chemical Reagents Co., Ltd. (Shanghai, China). Glucose, anhydrous ethanol, sodium borohydride, simvastatin, oleic acid, and dimethyl sulfoxide (DMSO) were purchased from Aladdin Biochemical Technology Co., Ltd. (Shanghai, China). Monosaccharide standards including Mannose (Man), Rhamnose (Rha), Glucuronic acid (GlcA), Galacturonic acid (GalA), Glucose (Glc), Galactose (Gal), Arabinose (Ara), and Fucose (Fuc) were purchased from Sigma Chemical Co. (St. Louis, MO, USA). Macroporous resin AB-8 was purchased from Macklin Biochemical Co., Ltd. (Shanghai, China). High-glucose Dulbecco’s Modified Eagle’s medium (DMEM) was purchased from Solarbio Science & Technology Co., Ltd. (Beijing, China). Penicillin-streptomycin was purchased from HyClone Laboratories, Inc. (Logan, Utah, USA). The RIPA lysis buffer was purchased from Sikejie Biotechnology Co., Ltd. (Jinan, Shandong, China). SREBP1-C antibody was purchased from Sangon Biotech Co., Ltd. (Shanghai, China). SIRT-1 and AMPK antibodies were purchased from Yakang Biotechnology Co., Ltd. (Wuhan, Hubei, China). Fetal bovine serum was purchased from Cyagen Biosciences Inc. (Wuhan, Hubei, China). Thiazolyl blue MTT agent was purchased from Lanjieke Information Technology Co., Ltd. (Hefei, Anhui, China).

### 2.2 Preparation of Pomegranate Peel Polysaccharide PPP-1

Pomegranate peels (100 g) were dried and ground into fine powder. The powder was refluxed with 95% ethanol at a ratio of 1:10 (g/mL) for 2.5 h. The resulting residue was collected and oven-dried at 85 °C. The pretreated powder was then extracted with hot water at a solid-to-liquid ratio of 1:20 (g/mL) at 90 °C for 1.5 h three times. The combined extracts were then treated with thermostable α-amylase (5000 U/mL, 95 °C, pH 6.5, 1 h) and glucoamylase (1000 U/mL, 60 °C, pH 4.5, 30 min). After enzymatic hydrolysis, the mixture was centrifuged at 4543*g* for 10 min. The supernatant was concentrated to approximately one-sixth of its original volume, and four volumes of anhydrous ethanol were added. The mixture was kept at 4 °C overnight. The precipitates were then collected, washed with ethanol, freeze-dried, and dissolved in deionized water. Proteins in the extract were removed using microporous resin AB-8. Finally, the solution was concentrated, dialyzed against distilled water (molecular weight cutoff of 3500 Da), and freeze-dried to obtain the crude pomegranate peel polysaccharide.

The crude polysaccharide fraction was dissolved and passed through a 0.45 μm membrane filter. The filtrate was then loaded onto a DEAE-FF anion exchange column (2 cmφ × 20 cm), which was eluted sequentially with deionized water and NaCl solution (0.1–0.5 mol/L). The eluted fractions were monitored using a phenol-sulfuric acid method.^18^ Fractions containing the same polysaccharide peaks were combined and further purified by Sephadex G-75 gel permeation chromatography (2.6 cmφ × 70 cm). The main fraction was collected, concentrated, and freeze-dried to obtain the polysaccharide fraction, designated as PPP-1.

### 2.3 Characterization of Polysaccharide PPP-1

#### 2.3.1 Preliminary Characterization of PPP-1

The molecular weight (*M*_w_) of PPP-1 was determined by high-performance liquid chromatography (HPLC; RCL1260; Santa Clara, CA, USA) equipped with an ELSD detector and a TSK G-6000 column using ultrapure water as eluent with an injection volume of 20 μL of sample at a flow rate of 0.6 mL/min.^19^ Dextrans (T-3, T-10, T-50, T500, T-1000, and T-2000) were used as standards. A total carbohydrate content was measured using the phenol-sulfuric acid method,^18^ while a protein content was determined by Bradford’s method.^20^ The phenolic content was measured by the Folin-Ciocalteu method.^21^ The uronic acid content was quantified by the sulfuric acid-carbazole method.^22^ The viscosity of PPP-1 solution was measured at various shear rates using a rotational rheometer (MCR302; Anton Paar GmbH; Graz, Styria, AT).

The presence of a triple-helix structure was confirmed by a Congo red assay. A PPP-1 polysaccharide solution (2 mL; 2.5 mg/mL) was added to a Congo Red solution (2 mL; 160 mM). NaOH solution (1 M) was then added stepwise so that the final NaOH concentration in the mixture increased gradually from 0 to 0.5 M. A full-wavelength UV scan (TU-1901; Purkinje, Beijing, China) was performed over the range 400–700 cm^–1^.

The FT-IR spectrum of PPP-1 was recorded using the KBr-pellet method.^23^ PPP-1 (5 mg) was thoroughly dried, ground with KBr powder (200 mg), and pressed into a pellet (1 mm thick). The spectrum was recorded with a spectrometer (Nicolet 6700, Thermo Fisher, Waltham, MA, USA) over the wavelength range of 4000 to 400 cm^–1^.

#### 2.3.2 Monosaccharide Composition Analysis

The monosaccharide composition of PPP-1 was determined using a precolumn derivatization method with 1-phenyl-3-methyl-5-pyrazolone (PMP), with slight modifications.^24^ PPP-1 (10 mg) was dissolved in 5 mL of 2.5 M trifluoroacetic acid (TFA) and transferred to a 15 mL screw-capped tube, purged with N_2_, sealed, and hydrolyzed at 100 °C for 12 h. After hydrolysis, the solvent was evaporated under reduced pressure at 60 °C, and methanol was repeatedly added to remove residual TFA until a neutral pH was achieved. The residue was dissolved in deionized water (1.2 mL). For derivatization, equal volumes of NaOH (0.3 M) and PMP (0.5 M) were added, and the mixture was heated at 70 °C for 1 h. Subsequently, the reaction was neutralized with an equal volume of HCl (0.3 M) to pH 7, and extracted three times with an equal volume of chloroform until the organic phase was clear. The aqueous phase was collected, filtered, and analyzed by HPLC with a diode-array detection (DAD) detector. Monosaccharide standards were also subjected to the same derivatization procedure. The HPLC analysis was performed using an Agilent 1260 system equipped with a DAD detector and a Waters XBridge C18 column (4.6 mmφ × 250 mm, 5 μm). The mobile phase consisted of ammonium acetate solution (0.2 M) and acetonitrile at a flow rate of 1.0 mL/min.

### 2.4 Effect of PPP-1 on Lipid Metabolism in Oleic Acid-Treated HepG2 Cells

#### 2.4.1 Cell Culture

HepG2 cells were obtained from the Cell Bank of the Chinese Academy of Sciences (Shanghai, China) and cultured in DMEM supplemented with 10% FBS and 1% penicillin–streptomycin–amphotericin B solution.^25^ Cells were maintained at 37 °C in a humidified incubator with 5% CO_2_.

#### 2.4.2 Optimum of Oleic Acid Concentration

HepG2 cells were seeded in 96-well plates and treated with 100 μL of oleic acid at different concentrations (0.1, 0.15, 0.2, 0.25, and 0.3 mM) for 24 h. After incubation, the culture medium was aspirated, and cell viability was assessed using the MTT assay following standard protocols.

#### 2.4.3 Cell Viability

Cell viability was assessed by an MTT assay.^25^ HepG2 cells were seeded into a 96-well plate and incubated with an optimal concentration of oleic acid. Subsequently, cells were treated with varying concentrations of PPP-1 (0, 50, 100, 200, 400, and 600 μg/mL) for an additional 24 h, with eight replicate wells per treatment group. After incubation, the medium was removed, and culture medium containing 0.5% MTT (100 μL) was added to each well. Following a 4-h incubation, the medium was discarded, and DMSO (100 μL) was added to dissolve the formazan crystals. Plates were gently shaken in the dark for 10 min, and the absorbance was measured at 570 nm with a microplate reader (Epoch-2, Biotek; Winooski, VT, USA).

#### 2.4.4 Measurement of Lipid Accumulation by Oil Red O Staining Assay

HepG2 cells were seeded into six-well plates and incubated for 24 h. Subsequently, lipid accumulation was induced by treating cells with the optimal concentration of oleic acid for 24 h. Cells in the treatment groups were incubated with various concentrations of PPP-1 (50, 200, and 600 μg/mL) or simvastatin (10 μM) for an additional 24 h. At the end of treatment, cells were washed twice with PBS and fixed with 4% paraformaldehyde for 30 min at room temperature. The fixed cells were rinsed with PBS and stained with a freshly prepared Oil Red O working solution for 30 min to visualize lipid droplets. Excess stain was removed by washing with distilled water. The stained lipid droplets were observed and imaged under a bright-field microscope (CKK41, Olympus; Shinjuku Ward, Tokyo, Japan). For the quantitative analysis, the dye retained in the cells was eluted with isopropanol, and absorbance was measured at 510 nm using a microplate reader (Epoch 2).^26^

#### 2.4.5 Biochemical Analysis

HepG2 cells were cultured with different concentrations of PPP-1 or simvastatin for 24 h. After treatment, the culture supernatants were collected to determine the activities of alanine aminotransferase (ALT) and aspartate aminotransferase (AST) using commercial assay kits according to the manufacturer’s instructions. The cells were then washed with PBS and lysed on ice for 30 min. The lysates were centrifuged at 12,000 rpm for 10 min at 4 °C, and the supernatants were collected to measure levels of total cholesterol (TC), triglycerides (TG), low-density lipoprotein cholesterol (LDL-C) and high-density lipoprotein cholesterol (HDL-C) using assay kits following the manufacturer’s protocols.

#### 2.4.6 The Effect of PPP-1 on the Expression of Related Genes

HepG2 cells were treated with blank medium, PPP-1 (at concentrations of 50, 200, and 600 μg/mL), or simvastatin for 24 h as described in Section 2.4.4. Total RNA was extracted using TRIzol reagent, and cDNA was synthesized via reverse transcription. Quantitative real-time PCR was performed to measure the mRNA expression levels of sterol regulatory element-binding protein 1c (SREBP-1c), fatty acid synthase (FAS), and sirtuin 1 (SIRT1), with actin serving as the internal reference gene. Relative gene expression was calculated using the 2^–△△CT^ method.^27^

#### 2.4.7 Western Blot Analysis

HepG2 cells were treated with blank medium, PPP-1 (at concentrations of 50, 200, and 600 μg/mL), or simvastatin for 24 h. After treatment as aforementioned, cells were washed with ice-cold PBS and lysed with RIPA lysis buffer (Beyotime) supplemented with protease and phosphatase inhibitors on ice for 30 min. The lysates were centrifuged at 8000*g* for 20 min at 4 °C, and the supernatants were collected. Protein concentrations were determined using a BCA assay (Solarbio; Beijing, China), and equal amounts of protein were separated by SDS-PAGE, transferred onto PVDF membranes, and subjected to immunoblotting with specific primary and secondary antibodies. Immunoreactive bands were visualized using an enhanced chemiluminescence detection system with an ECL kit (Beyotime), and band intensities were quantified using ImageJ software.

### 2.5 Effects of PPP-1 on Lipid Metabolism in High-Fat Diet-Fed Mice

#### 2.5.1 Animal Experiment

The C57BL/6J mice (20 ± 2 g) were purchased from the Experimental Animal Center of Anhui Medical University (Hefei, Anhui, China). Following a one-week acclimatization under controlled conditions (temperature 22 ± 2 °C, relative humidity 60%, 12 h light/dark cycle) with ad libitum access to standard chow and water, mice were randomly assigned into four groups (*n* = 10 per group): normal diet group (ND), high-fat diet group (HFD), normal diet + PPP-1 group (ND + PPP-1, 200 mg/kg/day), and high-fat diet + PPP-1 group (HFD + PPP-1, 200 mg/kg/day). PPP-1 administration was initiated concurrently with high-fat diet (HFD) feeding to evaluate its preventive effects on diet-induced metabolic dysfunction, rather than its therapeutic efficacy after disease establishment. PPP-1 was administered via daily oral gavage at a dose of 200 mg/kg body weight for nine consecutive weeks. The dosage of 200 mg/kg/day was selected based on previous studies^28^ demonstrating the efficacy of dietary polysaccharides in the range of 100–400 mg/kg/day in high-fat diet-induced metabolic disorder models,^29,30^ balancing biological activity and safety. Body weight and food intake were recorded weekly throughout the experiment. At the end of the intervention, fresh fecal samples were collected, freeze-dried, and weighed. Lipid extraction and fecal lipid content were determined gravimetrically to calculate lipid excretion.^31^ Following a 6 h fast, all mice were euthanized. Blood samples were collected, and tissues, including liver, epididymal fat, and cecal contents, were harvested, immediately frozen in liquid nitrogen, and stored at −80 °C for further analyses. All animal care and experimental protocols were conducted in accordance with the China Council on Animal Care and approved by the Animal Ethics Committee, Anhui University (IACUC­(AHU)-2024–018).

#### 2.5.2 Biochemical Indicator Analysis

Blood samples were centrifuged at 3000 rpm for 20 min at 4 °C to separate serum. Serum levels of TC, TG, HDL-C, and LDL-C were measured using commercially available biochemical assay kits according to the manufacturer’s instructions. Liver samples were weighed and homogenized. The homogenates were analyzed for biochemical indicators, including total bile acids (TBA), ALT, and AST, using commercial kits.

#### 2.5.3 Histological Analysis

For histopathological evaluation, liver, colon, and epididymal fat tissues were fixed in 4% paraformaldehyde, embedded in paraffin, sectioned, and stained with hematoxylin-eosin (H&E) to assess tissue morphology (CKK41, Olympus). Additionally, liver tissues were cryosectioned and stained with Oil Red O to visualize lipid accumulation under light microscopy (CKK41).

#### 2.5.4 Gut Microbiota Analysis

Total DNA was extracted from the cecal contents stored at −80 °C using a commercial DNA extraction kit according to the manufacturer’s protocol. The V3–V4 regions of bacterial 16S rRNA genes were amplified by PCR (A200, LongGene; HangZhou, Zhejiang, China) and high-throughput sequencing was performed on an Illumina MiSeq PE300 system (Illumina; San Diego, CA, USA). Sequence data were analyzed for microbial community structure and composition.

#### 2.5.5 Untargeted Metabolomics Analysis

Untargeted metabolomic profiling of mouse cecal contents was conducted on Quadrupole time-of-flight high resolution mass spectrometry (Triple TOF 6600, AB SCIEX; Redwood City, CA, USA). Metabolites were annotated using the Human Metabolome Database (HMDB). Principal component analysis (PCA) was performed to visualize group separation.^32^ Differential metabolites were identified based on VIP > 1 and *P* < 0.05, and pathway enrichment analysis was conducted using the Kyoto Encyclopedia of Genes and Genomes (KEGG) database to identify key metabolic pathways modulated by PPP-1 treatment.

### 2.6 Statistical Analysis

Statistical analysis was performed using one-way ANOVA in GraphPad Prism version 9.0.0 (GraphPad; San Diego, CA, USA). Data are presented as mean ± standard deviation (SD), and *P* values <0.05 were considered statistically significant.

## 3 Results

### 3.1 Physicochemical Characterization of PPP-1

A new purified polysaccharide, designated PPP-1, was successfully isolated from pomegranate peel via hot-water extraction and multistep chromatography. It was obtained as a white powder with an overall yield of 5.24% from the crude polysaccharide extract. Chemical analysis revealed a total carbohydrate content of 96.7%, with no detectable protein or phenolic components. The absence of uronic acid confirmed that PPP-1 is a neutral polysaccharide with no starch characteristics, as supported by a negative iodine–potassium iodide test.

High-performance size-exclusion chromatography (HPSEC) analysis showed a single, symmetrical peak, suggesting that PPP-1 is a homogeneous fraction (Figure 1A). Based on a calibration curve derived from dextran standards (log *M*_w_ = −0.2422*t* + 8.7833, *R* ^2^ = 0.998), the weight-average molecular weight (*M*_w_) of PPP-1 was determined to be 1.5 × 10^4^ Da. The monosaccharide composition was characterized by HPLC analysis following PMP derivatization (Figure 1B,C). The results showed that PPP-1 is composed of glucose, mannose, arabinose, and galactose in a molar ratio of 75.24:3.33:1.00:1.18. This glucose-rich composition supports the classification of PPP-1 as a glucan.

**Figure 1:**
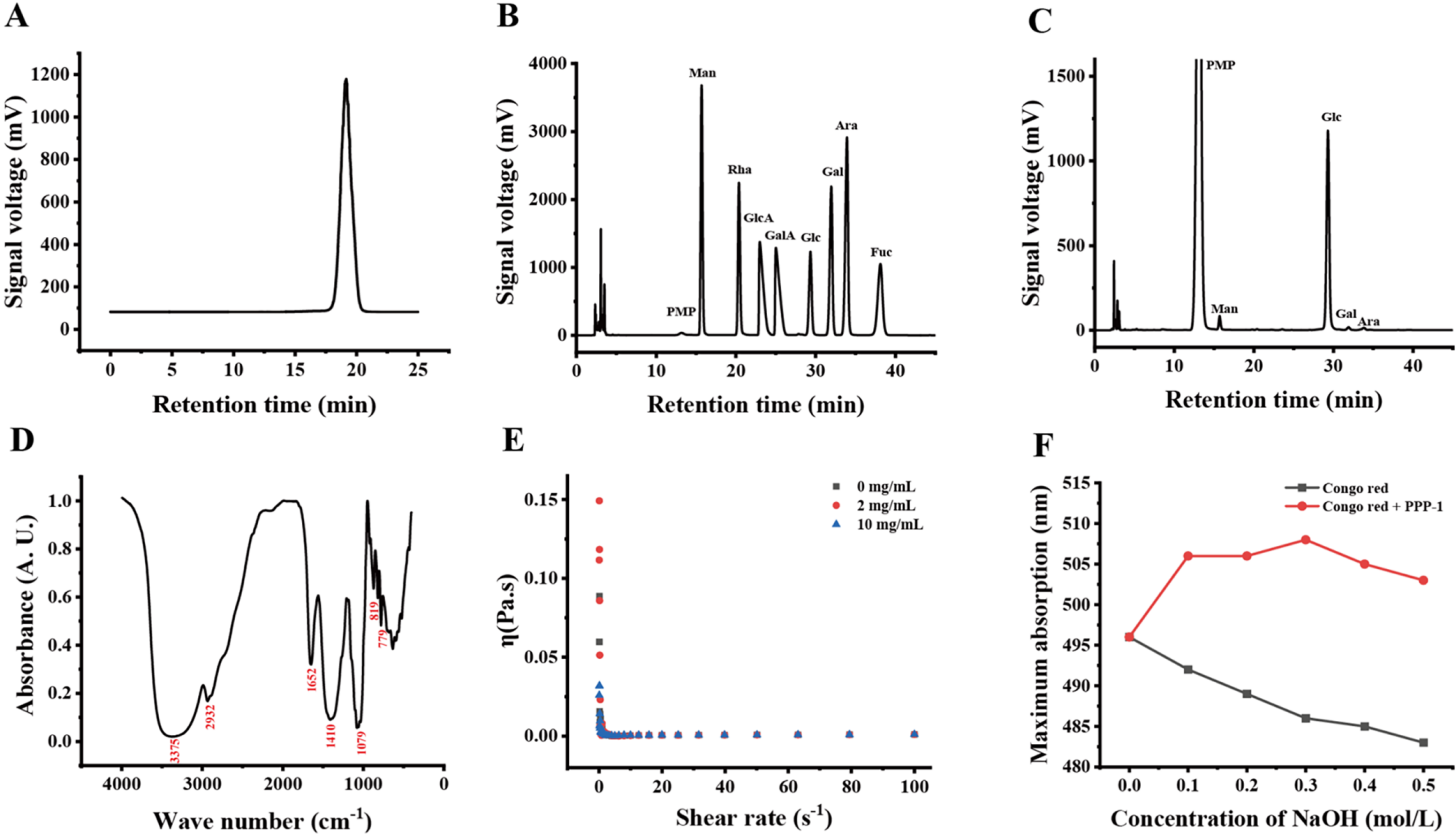
Physicochemical and structural characterization of the pomegranate peel polysaccharide PPP-1. (A) HPLC profile of PPP-1. (B, C) HPLC chromatograms for monosaccharide composition analysis of the mixed standards and the acid hydrolysate of PPP-1. (D) FTIR spectrum of PPP-1. (E) Viscosity-shear rate curve of PPP-1. (F) Congo red test of PPP-1.

The FT-IR spectrum of PPP-1 displayed characteristic absorption bands of polysaccharides (Figure 1D). A broad, strong band at 3375 cm^–1^ was attributed to the O–H stretching vibrations, while the peak at 2932 cm^–1^ corresponded to C–H stretching. The absorption at 1652 cm^–1^ showed the presence of bound water. Key peaks in the fingerprint region, including those at 1410 and 1079 cm^–1^, were assigned to C–O stretching vibrations within the pyranose rings. Furthermore, characteristic bands at 819 and 779 cm^–1^ suggested the presence of both α- and β-type glycosidic linkages in the polysaccharide backbone.

The macromolecular properties of PPP-1 in solution were also investigated. Rheological analysis showed that the apparent viscosity of the PPP-1 solution remained constant with increasing shear rates, which is a characteristic of a low-viscosity polysaccharide (Figure 1E). The advanced conformation of PPP-1 was explored using a Congo red assay. The maximum absorption wavelength of the PPP-1-Congo red complex exhibited a significant red shift compared to Congo red alone, providing strong evidence that PPP-1 possesses a stable triple-helix conformation in aqueous solution (Figure 1F). Collectively, the results from monosaccharide composition, molecular weight determination, FT-IR spectroscopy, and Congo red assay indicate that PPP-1 is a homogeneous, glucose-rich neutral glucan with both α- and β-glycosidic linkages and a stable triple-helix conformation. Although detailed linkage patterns and sequence information require further elucidation, these complementary analyses provide a reliable structural basis for preliminary characterization of PPP-1.

### 3.2 PPP-1 Ameliorates Oleic Acid-Induced Lipid Dysregulation in HepG2 Cells

#### 3.2.1 PPP-1 Attenuates Intracellular Lipid Accumulation

To model hepatic steatosis *in vitro*, HepG2 cells were treated with oleic acid (OA).^33^ An optimal concentration of 0.25 mM OA was selected, as it induced lipid accumulation while maintaining acceptable cell viability (78%) (Figure 2A). Prior to evaluating its efficacy, we confirmed that PPP-1 exhibited no significant cytotoxicity at concentrations up to 600 μg/mL (Figure 2B). Based on these results, subsequent experiments were conducted at low (50 μg/mL), medium (200 μg/mL), and high (600 μg/mL) concentrations of PPP-1.

**Figure 2:**
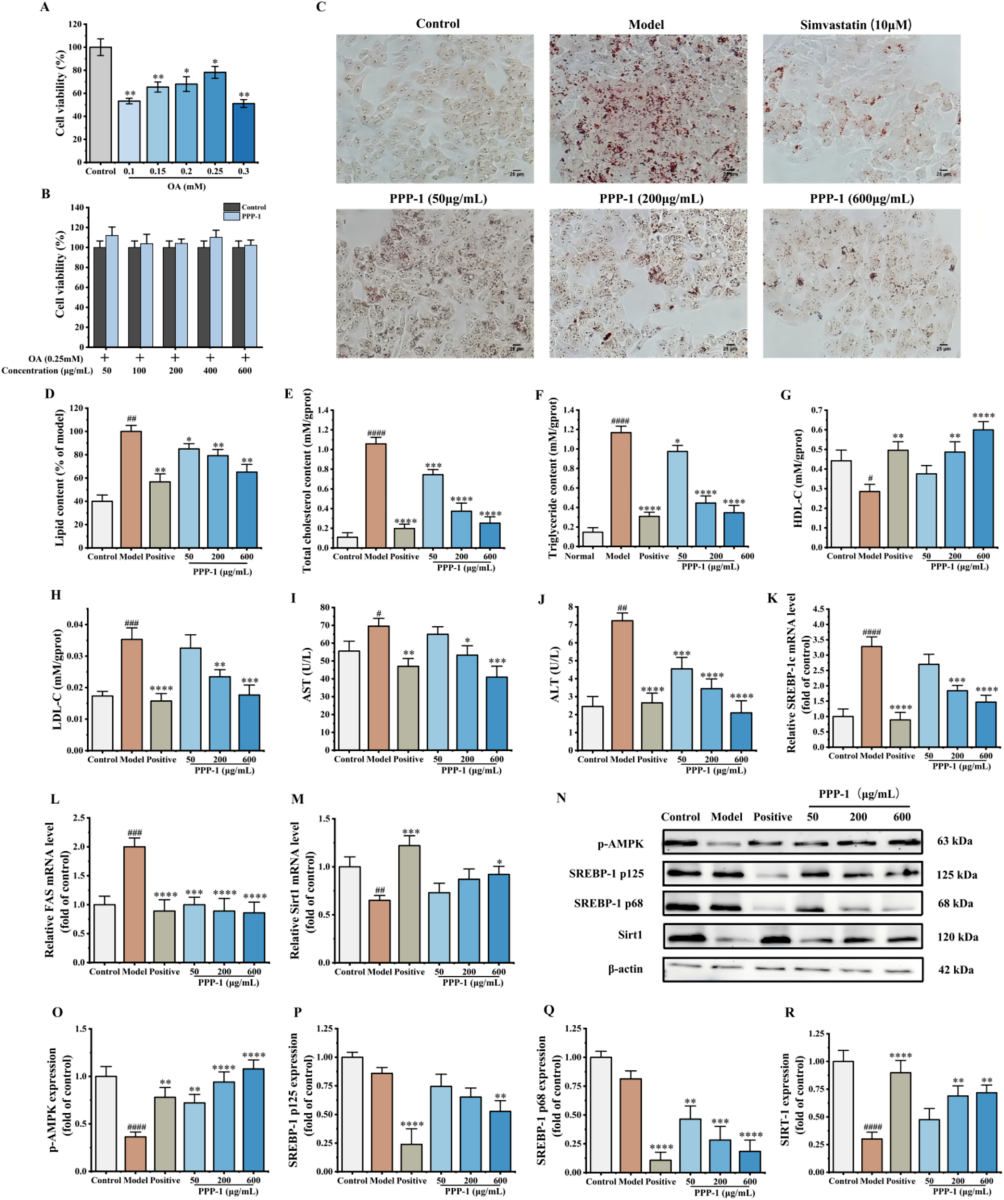
PPP-1 ameliorates OA-induced steatosis and metabolic dysfunction in HepG2 cells. (A) Viability of HepG2 cells after treatment with different concentrations of OA. (B) Cytotoxicity assessment of PPP-1 on HepG2 cells. (C, D) Representative images and quantitative analysis of intracellular lipid accumulation visualized by Oil Red O staining. (E–H) Intracellular levels of TG, TC, HDL-C, and LDL-C. (I, J) Levels of AST and ALT in the cell culture supernatant. (K–M) Relative mRNA expression of lipogenic genes SREBP-1c, FAS, and the regulator SIRT1. (N–R) Representative Western blot images and quantification analysis for p-AMPK, SREBP-1c, and SIRT1 proteins. Data are expressed as mean ± standard deviation (SD). ^#^ *P* < 0.05, ^##^ *P* < 0.01, ^###^ *P* < 0.001, and ^####^ *P* < 0.0001 vs the control group, respectively. *P < 0.05, **P < 0.01, ***P < 0.001, and ****P < 0.0001 vs the OA-treated model group, respectively.

As visualized by Oil Red O staining,^34^ OA treatment induced the formation of numerous large lipid droplets compared to the control group. Treatment with PPP-1 led to a clear, concentration-dependent reduction in both the size and number of these droplets (Figure 2C). Quantitative analysis confirmed this observation, showing that high-dose (600 μg/mL) PPP-1 reduced the total lipid area by 34.8% (*P* < 0.01), compared to the OA-treated model group, supporting its potent ability to mitigate hepatocyte lipid deposition (Figure 2D).

#### 3.2.2 PPP-1 Restores Key Markers of Lipid Metabolism and Reduces Hepatocyte Injury

Consistent with the visual evidence of lipid reduction, PPP-1 also modulated the biochemical markers of lipid metabolism. OA induction led to a rise in intracellular TC and TG levels. Treatment with PPP-1 concentration-dependently reversed these increases. Notably, PPP-1 at 600 μg/mL reduced TC and TG levels by 76.2 and 70.4%, respectively, which is comparable to that of the positive control, simvastatin (Figure 2E,F).

Furthermore, PPP-1 modulated cholesterol transport markers^35^ by increasing the level of HDL-C by 2.1-fold while decreasing LDL-C by 50% at the highest concentration (Figure 2G,H). PPP-1 also exhibited cytoprotective effects by attenuating the OA-induced leakage of ALT and AST, two sensitive indicators of liver cell damage, in a concentration-dependent manner (*P* < 0.05) (Figure 2I,J).

#### 3.2.3 PPP-1 Modulates the SIRT1/AMPK Lipogenesis Pathway

Fatty acid synthase (FAS) and sterol regulatory element-binding protein-1c (SREBP-1c) play central roles in hepatocellular lipid synthesis: FAS catalyzes the de novo formation of long-chain fatty acids, while SREBP-1c upregulates FAS and other lipogenic genes, promoting triglyceride accumulation.^36,37^ Conversely, SIRT1, a NAD^+^-dependent deacetylase, is known to inhibit SREBP-1c via deacetylation, thus suppressing lipogenic gene expression and promoting fatty acid oxidation.^38^ To investigate the molecular basis for its lipid-lowering effects, we first examined the transcriptional regulation of these key genes. The treatment of OA upregulated the mRNA levels of the lipogenic genes SREBP-1c and FAS. Co-treatment with PPP-1 counteracted this effect by inducing concentration-dependent suppression of SREBP-1c and FAS. At the 600 μg/mL, PPP-1 reduced SREBP-1c and FAS mRNA levels by 2.23-fold and 2.32-fold, respectively, compared to the OA model group (Figure 2K,L). Moreover, PPP-1 treatment led to a concentration-dependent increase in the mRNA expression of the upstream regulator SIRT1 (Figure 2M).

These transcriptional changes were subsequently supported at the protein level by Western blot analysis (Figure 2N–R). Consistent with the qPCR data, PPP-1 treatment suppressed the protein expression of SREBP-1c. Notably, PPP-1 targeted the upstream regulators of the pathway. While OA treatment reduced SIRT1 protein levels by approximately 70%, this effect was significantly rescued by PPP-1 (at 600 μg/mL), which restored SIRT1 expression by 58.2%. Concurrently, PPP-1 increased the phosphorylation of AMPK, a key cellular energy sensor, by 2.97-fold. Collectively, these results suggest that PPP-1 mitigated lipid accumulation by activating the SIRT1/AMPK signaling axis, leading to the suppression of SREBP-1c-mediated lipogenesis.

### 3.3 PPP-1 Ameliorates HFD-Induced Lipid Dysfunctions *In Vivo*

#### 3.3.1 PPP-1 Prevents Obesity and Corrects Dyslipidemia

To evaluate the *in vivo* efficacy of PPP-1, C57BL/6J mice were fed a high-fat diet (HFD) with or without PPP-1 supplementation (200 mg/kg/day) for 9 weeks (Figure 3A). The HFD induced a significant increase in body weight and visceral fat. However, coadministration of PPP-1 potently counteracted these effects, reducing body weight gain by 42.1% and epididymal fat mass by 41.5% compared to the HFD group (*P* < 0.0001) (Figure 3B,C). Histological analysis of adipose tissue confirmed these findings, revealing that PPP-1 suppressed HFD-induced adipocyte hypertrophy by 57.1% (Figure 3D,E). Notably, PPP-1 produced no measurable changes in body weight or adiposity in mice fed a normal diet, suggesting that its actions are selectively triggered by metabolic perturbations associated with high-fat feeding. Moreover, the antiobesity effects of PPP-1 were not attributed to changes in appetite or nutrient absorption. Daily energy intake did not differ among the groups, and PPP-1 supplementation did not elevate fecal lipid excretion (Figure 3G,H). This suggests that the metabolic benefits of PPP-1 arise from its regulation of lipid metabolism, rather than from altered digestive efficiency or impaired lipid absorption.

**Figure 3:**
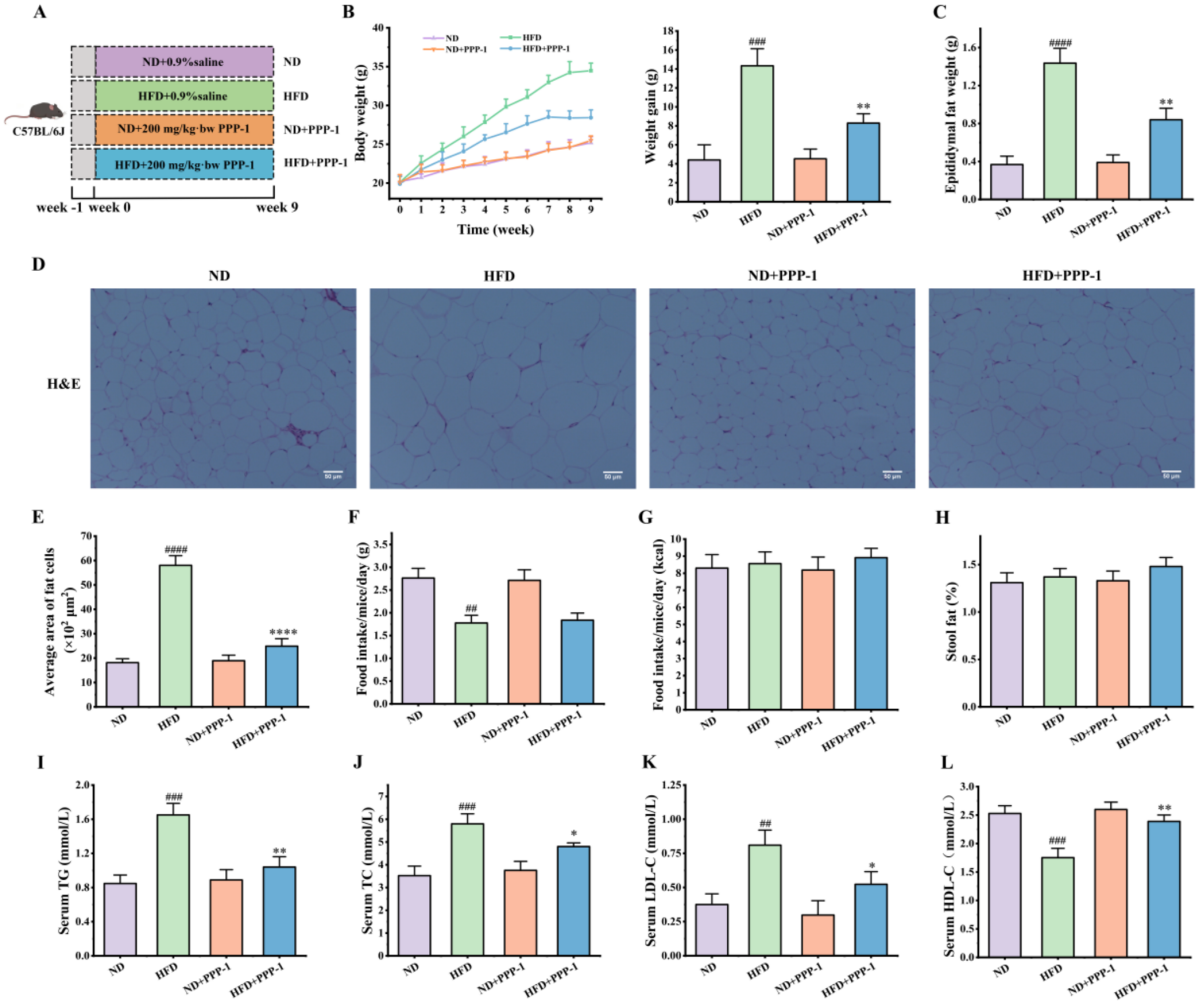
PPP-1 alleviates HFD-induced obesity and dyslipidemia in mice. (A) Schematic diagram of the *in vivo* experimental design. (B) Weekly body weight changes. (C) Epididymal adipose tissue weight. (D) Representative images of H&E stained epididymal adipose tissue. (E) Quantification of average adipocyte area. (F, G) Average daily food and energy intake per mouse. (H) Fecal lipid excretion. (I–L) Serum levels of TG, TC, LDL-C, and HDL-C. Data are expressed as mean ± standard deviation (SD). ^##^ *P* < 0.01, ^###^ *P* < 0.001, and ^####^ *P* < 0.0001 vs ND group. *P < 0.05, **P < 0.01, and ****P < 0.0001 vs HFD group.

Consistent with the well-documented effects of a high-fat diet, which elevates TC, TG, and LDL-C while lowering HDL-C,^39^ the HFD group displayed marked dyslipidemia compared with the ND group (Figure 3I–L). PPP-1 supplementation ameliorated these abnormalities, reducing TC by 17.2%, TG by 37.0%, and LDL-C by 35.4%, while increasing HDL-C by 36.4% (all *P* < 0.05). These improvements align with the observed reductions in adiposity and demonstrate PPP-1’s capacity to counter HFD-induced dyslipidemia.

#### 3.3.2 PPP-1 Alleviates Hepatic Steatosis and Liver Injury in HFD-Fed Mice

An imbalance in lipid metabolism results in increased triglyceride storage within adipose depots and promotes ectopic lipid accumulation in nonadipose tissues, particularly the liver, where it contributes to steatosis.^40^ Metabolic disorders induced by HFD are known to trigger hepatic steatosis,^41^ which can progress to severe liver diseases.^42^

Ectopic lipid deposition in the liver is a key feature of HFD-induced metabolic syndrome. While the HFD caused a trend toward increased liver weight, PPP-1 supplementation improved the liver’s metabolic profile. It markedly reduced the hepatic accumulation of TG, TC, and LDL-C, and lowered TBA levels, while increasing hepatic HDL-C (Figure 4B–F). This improvement in hepatic lipid homeostasis was accompanied by hepatoprotective effects. The HFD caused a sharp rise in the serum levels of ALT and AST, which are key indicators of liver damage.^43^ PPP-1 supplementation lowered these enzymes by 27.9 and 32.1%, respectively, supporting its ability to preserve liver function (Figure 4G,H). Histological examination of liver tissue provided visual confirmation of these results. The H&E and Oil Red O stainings revealed severe micro- and macrovesicular steatosis in the HFD group, characterized by large lipid vacuoles and disrupted cellular architecture. In contrast, the livers of PPP-1-treated mice showed substantially reduced lipid droplet accumulation and well-preserved hepatocyte morphology, supporting its antisteatotic activity (Figure 4I,J).

**Figure 4:**
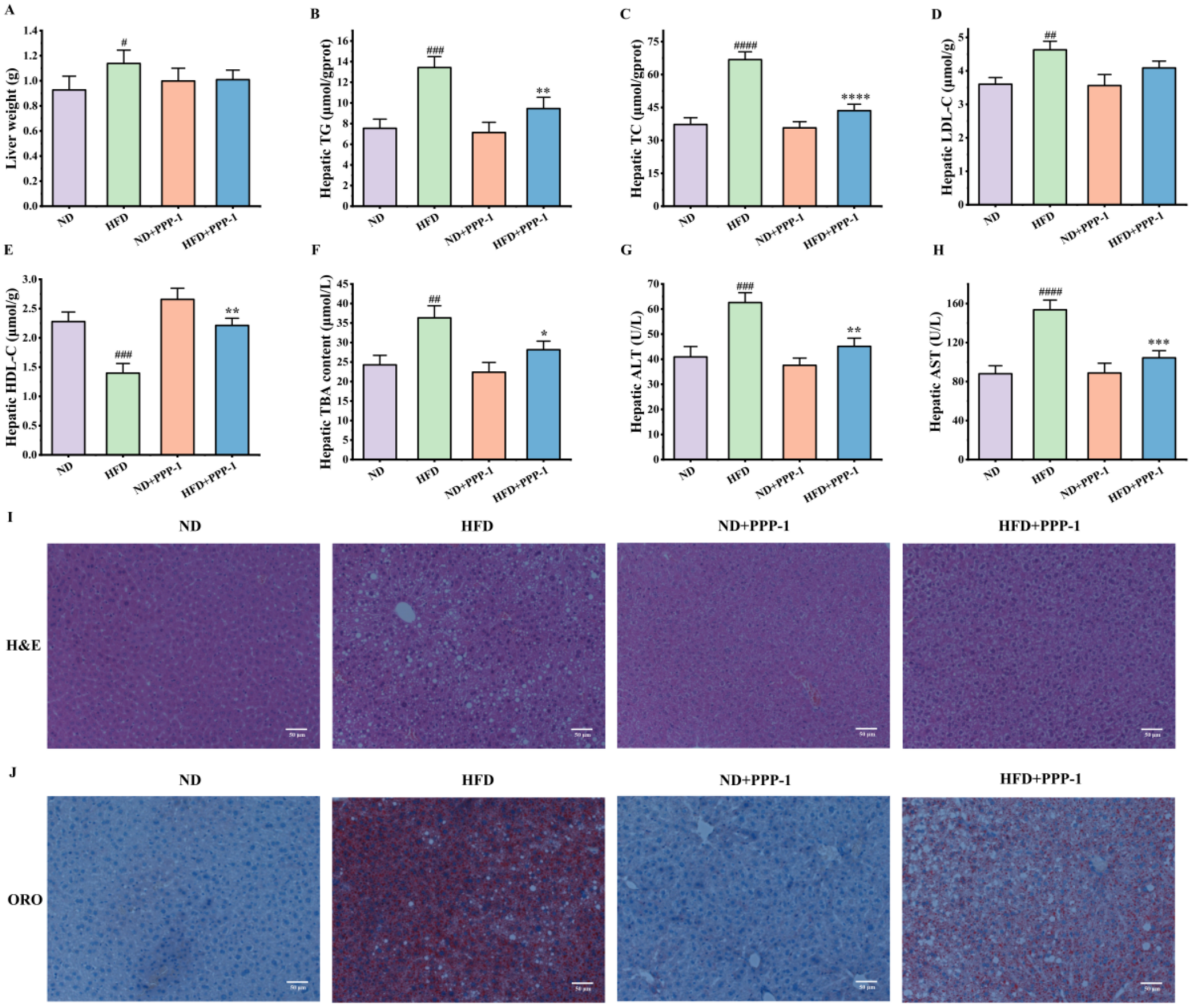
PPP-1 protects against HFD-induced hepatic steatosis and liver injury. (A) Liver weight. (B–F) Hepatic levels of TG, TC, LDL-C, HDL-C, and TBA. (G, H) Hepatic levels of AST and ALT. (I) Representative images of H&E-stained liver sections. (J) Representative images of ORO-stained liver sections. Data are expressed as mean ± standard deviation (SD). ^#^ *P* < 0.05, ^##^ *P* < 0.01, ^###^ *P* < 0.001, and ^####^ *P* < 0.0001 vs ND group. *P < 0.05, **P < 0.01, ***P < 0.001, and ****P < 0.0001 vs HFD group.

#### 3.3.3 PPP-1 Reshapes the Gut Microbiota Composition in HFD-Fed Mice

Given the link between gut dysbiosis and metabolic disease, we investigated the effect of PPP-1 on gut microbiota. OTU clustering at 97% similarity revealed distinct microbial shifts across the four groups. Compared with the ND group, the OTU count decreased in the ND + PPP-1, HFD, and HFD + PPP-1 groups (Figure 5A). Notably, 1384 OTUs were exclusive to the ND group relative to HFD, while 609 and 1441 OTUs were unique to the HFD + PPP-1 and ND + PPP-1 groups, respectively. These findings highlight both shared and group-specific microbial populations. α-diversity metrics indicated that the HFD tended to reduce microbial richness and diversity, an effect that was not significantly altered by PPP-1 (Figure 5B,C). However, β-diversity analysis revealed that PPP-1 induced a distinct shift in the overall microbial community structure, separating its profile from both the ND and HFD clusters (Figure 5D). This suggests that PPP-1 selectively modulated specific microbial taxa rather than a broad, nonspecific change.

**Figure 5:**
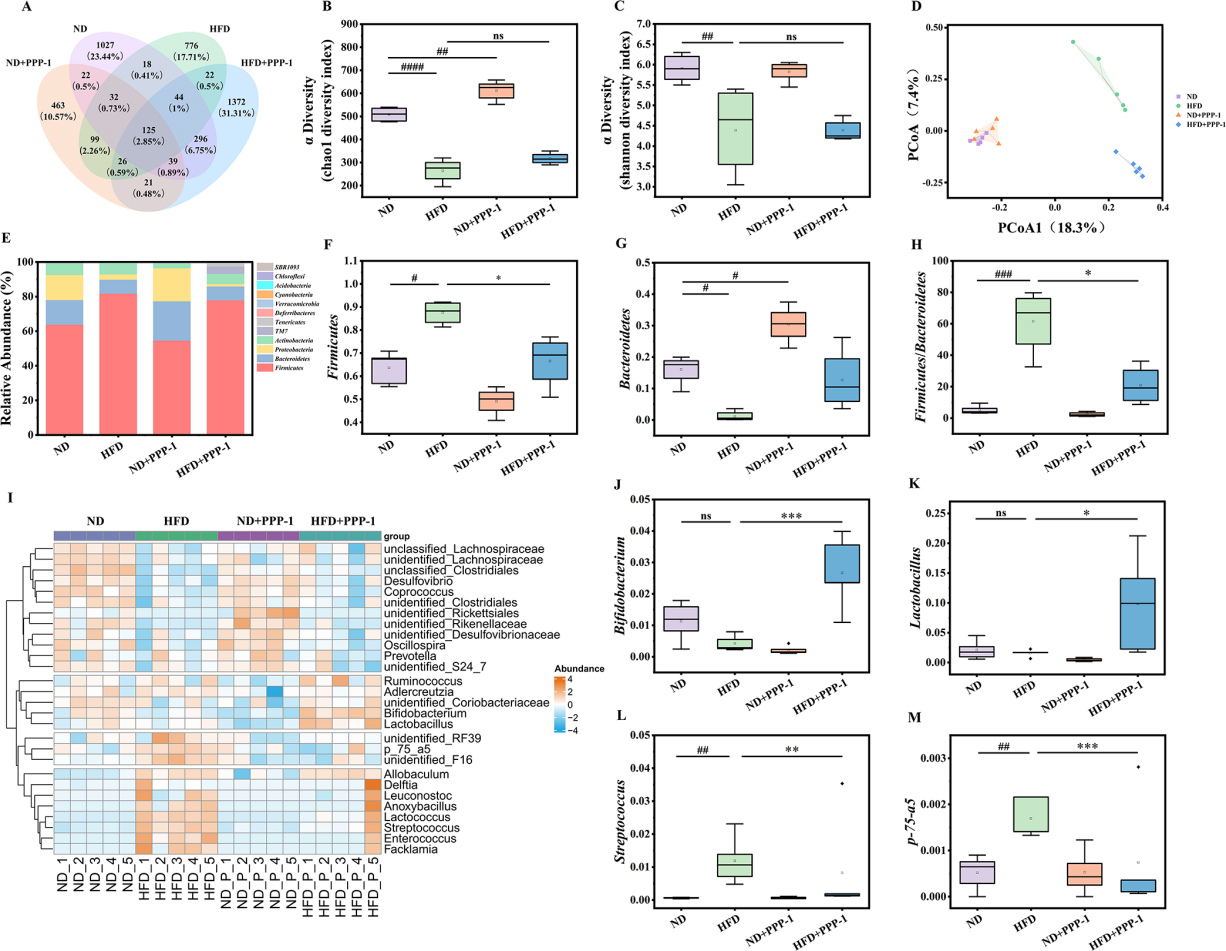
PPP-1 modulates the composition and structure of the gut microbiota in HFD-fed mice. (A) Venn diagram of OTUs across the different groups. (B, C) α diversity analysis using the Chao1 and Shannon indices. (D) Principal component analysis (PCA) plot of β diversity. (E) Relative abundance of gut microbiota at the phylum level. (F–H) Relative abundance of *Firmicutes*, *Bacteroidetes*, and the F/B ratio. (I) Heatmap illustrating the relative abundance of the genera. (J–M) Relative abundance of key differential genera. Data are expressed as mean ± standard deviation (SD). ^#^ *P* < 0.05, ^##^ *P* < 0.01, ^###^ *P* < 0.001, and ^####^ *P* < 0.0001 vs ND group. *P < 0.05, **P < 0.01, ***P < 0.001 vs HFD group.

PPP-1 supplementation modulated several key markers of obesity-associated dysbiosis in HFD-fed mice. At the phylum level, it lowered the F/B ratio (*P* < 0.05) by reducing *Firmicutes* (by 17.9%) and increasing *Bacteroidetes* (by 6.2%), which is a hallmark imbalance linked to obesity^44,45^ (Figure 5E–H). At the genus level, PPP-1 promoted beneficial bacteria, notably *Lactobacillus* and *Bifidobacterium*, while suppressing the HFD-induced bloom of genera like *p-75-a5* and *Streptococcus* (Figure 5I–M). These changes suggest that PPP-1 fosters a gut microbial profile more conducive to metabolic health.

#### 3.3.4 PPP-1 Modulates Cecal Metabolomic Profiles in HFD-Fed Mice

To understand the functional consequences of the altered microbiota, untargeted metabolomics was performed on cecal contents. Principal Component Analysis (PCA) revealed that the metabolic profile of the HFD group was distinct from the other groups, and PPP-1 treatment led to a shift in this profile (Figure 6A), aligning with the diet’s known metabolic impact.^46^

**Figure 6:**
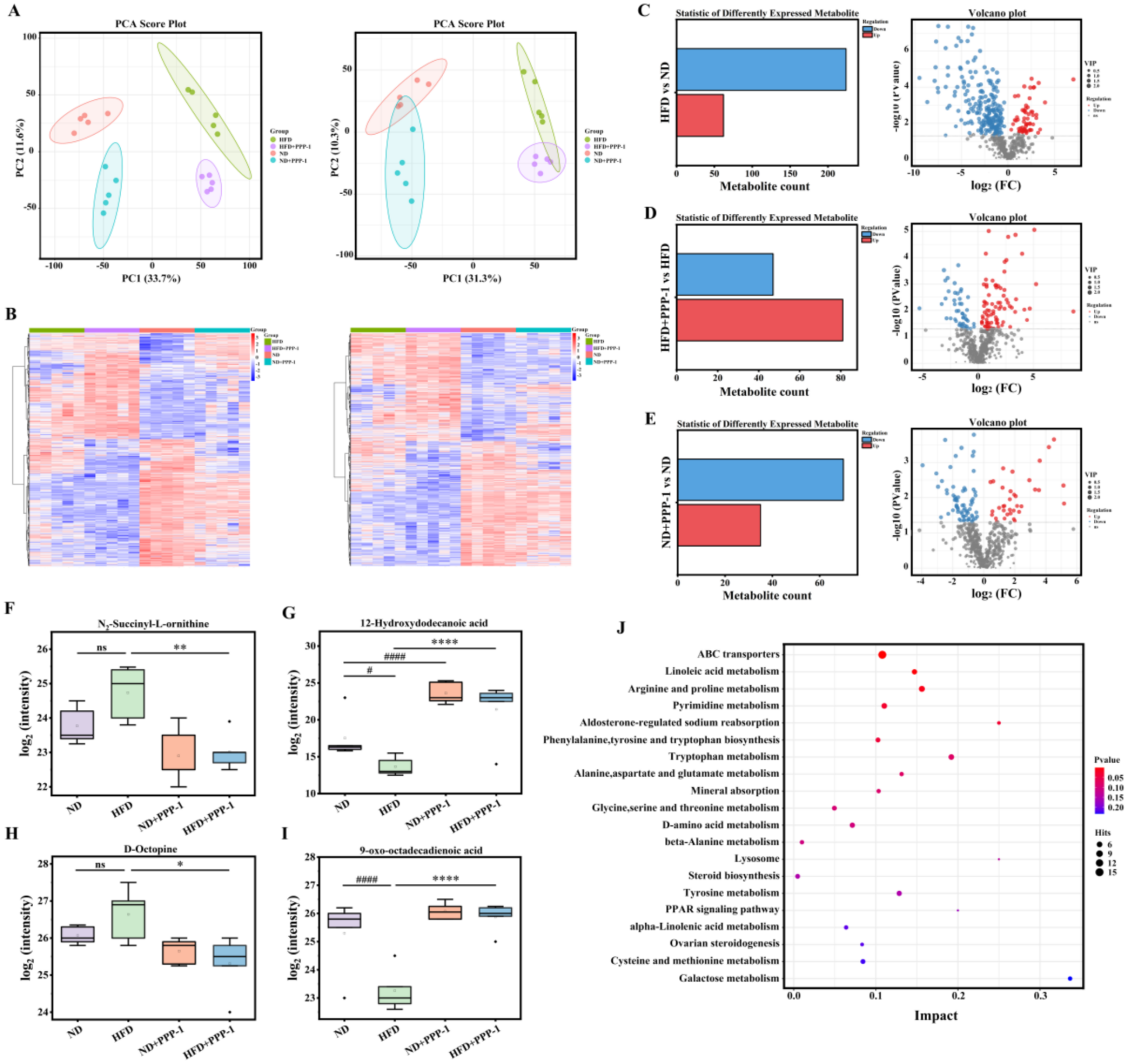
Cecal content metabolomics reveals the effect of PPP-1 on HFD-induced metabolic disturbances. (A) PCA score plots from untargeted metabolomics analysis in both positive (ESI+) and negative (ESI-) ionization modes. (B) Heatmap of differential metabolites identified in ESI+ and ESI- mode. (C–E) Volcano plots illustrating differential metabolites between comparison groups. (F–I) Box plots showing the relative abundance of key differential metabolites related to lipid metabolism. (J) KEGG pathway enrichment analysis of the differential metabolites. ^#^ *P* < 0.05, and ^####^ *P* < 0.0001 vs ND group. *P < 0.05, **P < 0.01, and ****P < 0.0001 vs HFD group.

A total of 10321 and 9843 metabolites were detected in the positive and negative ion modes, respectively, identified as significantly altered (VIP > 1, *P* < 0.05; 464 and 3048, respectively) (Figure 6B). The HFD itself induced widespread dysregulation, altering 286 metabolites (62 up and 224 down) involved in pathways for carboxylic acids, fatty acyls, and steroids (Figure 6C). PPP-1 supplementation reversed several of these HFD-induced disturbances. Compared to the HFD group, PPP-1 altered 128 metabolites (81 upregulated and 47 downregulated), particularly those involved in amino acid, carbohydrate, and fatty acid metabolism (Figure 6D). In the ND + PPP-1 group, PPP-1 influenced 35 upregulated and 70 downregulated metabolites (Figure 6E). Notably, PPP-1 corrected HFD-driven changes in key metabolites (Figure 6F–I): it normalized N_2_-succinyl-l-ornithine, restored depleted 12-hydroxydodecanoic acid and 9-oxo-octadecadienoic acid, and reduced the HFD-elevated D-Octopine.

The KEGG enrichment analysis confirmed these findings as PPP-1 significantly modulated 20 metabolic pathways (*P* < 0.05) compared to the HFD group. Key modulated pathways included ABC transporter systems, linoleic acid metabolism, and aromatic amino acid synthesis (Figure 6J). The modulation of these pathways, which play key roles in lipid transport, bile acid homeostasis, and inflammatory signaling, provides a mechanistic basis for the marked correction of HFD-induced dyslipidemia seen with PPP-1 treatment.

## 4 Discussion

The modern high-fat diet is a primary driver of metabolic disease, creating a state of chronic metabolic stress that promotes hepatic triglyceride synthesis, impairs fatty acid β-oxidation, and elevates circulating lipids. These disruptions culminate in obesity, hepatic steatosis, and insulin resistance, which are foundational to metabolic syndrome and cardiovascular disease.^47,48^ Therefore, identifying natural interventions that can restore lipid homeostasis is of paramount practical importance.

Pomegranate peel is a well-known reservoir of bioactive ingredients, particularly polysaccharides. Most reported examples are acidic, pectic-type heteropolysaccharides rich in galacturonic acid.^12,13^ While a neutral galactomannan polysaccharide has also been isolated from pomegranate,^14^ the present study identifies a novel glucose-rich glucan (PPP-1) characterized by α-/β-pyranose linkages and a triple-helix conformation. Although crude pomegranate extracts have previously shown lipid-lowering potential,^49^ the present study provides the first evidence that a purified and structurally defined polysaccharide fraction, PPP-1, acts as a promising metabolic regulator.

It is important to note that, consistent with the preventive focus of this study, PPP-1 was administered concurrently with HFD feeding to model a nutritional intervention rather than a therapeutic regimen. Similar experimental designs without pharmacological comparators are commonly adopted in studies of dietary polysaccharides and prebiotics.^50,51^ Nevertheless, we acknowledge that the absence of a drug-based positive control limits direct comparison with established lipid-lowering agents. Future studies incorporating pharmacological comparators will be valuable for further evaluation of PPP-1’s efficacy.

Biologically, the lipid-lowering and antiobesity effects observed here are consistent with previous reports on polysaccharides derived from *Auricularia auricula* ^52^ and *Platycodonis radix*,^53^ which similarly improved serum lipid profiles, liver function, and adiposity, ultimately contributing to antiobesity effects in HFD-fed mice. However, compared to these studies, PPP-1 is structurally distinct as a neutral glucan with a triple-helix conformation, and our study further extends these observations by integrating microbiota and metabolomics analyses to elucidate the gut–metabolite–liver axis.

Mechanistically, our cellular model demonstrates that PPP-1 modulated the SIRT1/AMPK signaling axis in hepatocytes, leading to increased AMPK phosphorylation and suppression of SREBP-1, a key transcriptional regulator of lipogenesis.^54^ By inhibiting SREBP-1 activation and its downstream transcriptional program, PPP-1 effectively reduces hepatic lipid accumulation. However, given the relatively large molecular weight of PPP-1 (c.a. 1.5 × 10^4^ Da), direct cellular uptake is unlikely. Therefore, the observed *in vitro* effects likely reflect interactions with cell surface receptors or indirect signaling pathways rather than intracellular penetration. In the animal model, PPP-1 is more likely to exert its metabolic effects through the gut–liver axis rather than direct cellular uptake due to its macromolecular nature. As an indigestible polysaccharide, PPP-1 can be metabolized by gut microbiota into bioactive metabolites, including fatty acid-derived signaling molecules, which subsequently regulate hepatic lipid metabolism. Consistent with this mechanism, PPP-1 significantly increased levels of 9-oxo-octadecadienoic acid and 12-hydroxydodecanoic acid, metabolites known to act as agonists of the PPAR signaling pathway (enriched in Figure 6J), which cooperates with SIRT1 to promote fatty acid β-oxidation. The marked reduction in hepatic triglyceride accumulation, together with the enrichment of pathways such as “ABC transporters” and “linoleic acid metabolism”, provides complementary evidence of suppressed de novo lipogenesis, consistent with the SREBP-1c inhibition observed *in vitro*. The strong concordance between phenotypic outcomes, cellular signaling, and systemic metabolic remodeling supports a biologically plausible mechanism of action for PPP-1.

In addition to its direct effects on hepatic lipid metabolism, PPP-1 also engages the gut–liver axis, representing a second major mechanism of action. The gut microbiota is intimately involved in metabolic homeostasis, and indigestible polysaccharides like PPP-1 serve as potent modulators of microbial composition and function. Our results showed that PPP-1 corrected HFD-induced dysbiosis, highlighted by the normalization of the F/B ratio. A high F/B ratio is a well-established biomarker for obesity, linked to increased energy harvesting efficiency from the diet.^55^ Thus, its reduction by PPP-1 signifies a foundational shift toward a leaner, healthier microbial profile. Furthermore, reduced populations of *Firmicutes* can alter the pool of secondary bile acids, a mechanism known to ameliorate HFD-induced lipid metabolism disorders.^56^ This gut remodeling was further supported by the enrichment of specific beneficial genera. PPP-1 reversed the HFD-induced depletion of *Lactobacillus* and *Bifidobacterium*, two probiotic genera known to alleviate fat accumulation.^57^ Correlation analysis confirmed their protective role, showing significant negative associations with liver weight, epididymal fat, and serum TC/TG (Figure 7). These correlations suggest that PPP-1-mediated enrichment of beneficial taxa may contribute to improved lipid metabolism through modulation of host metabolic and inflammatory pathways. Conversely, PPP-1 significantly suppressed *Streptococcus* and the pro-inflammatory genus p-75-a5. *Streptococcus* abundance was positively correlated with most metabolic risk markers and negatively with HDL-C (Figure 7), consistent with its known links to hepatic inflammation and metabolic dysfunction-associated steatohepatitis.^58^ Similarly, *p-75-a5* has been reported to correlate with HOMA-IR and lipopolysaccharide production,^59^ suggesting that their reduction contributes to the alleviated systemic inflammation observed here.

**Figure 7:**
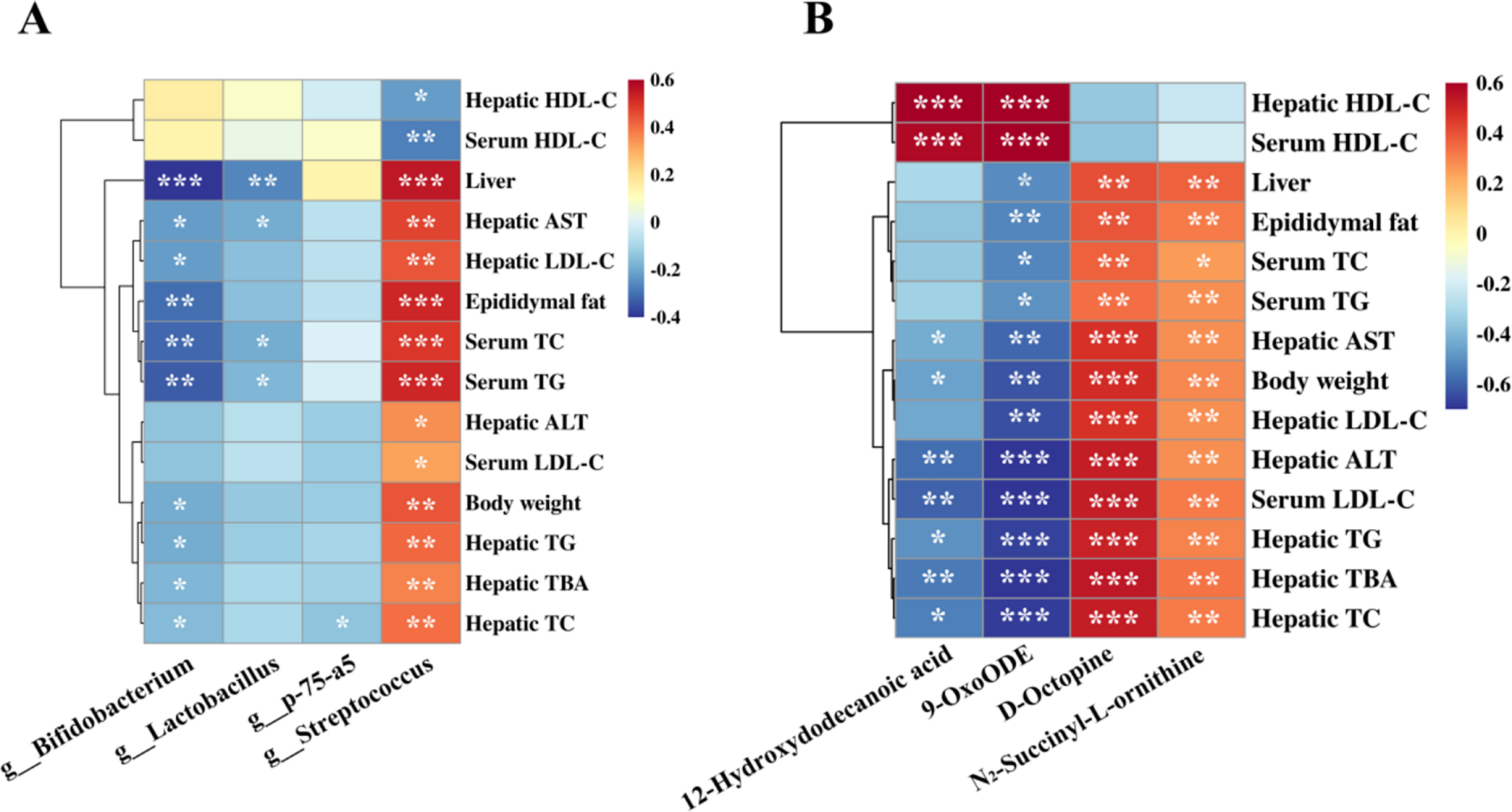
Multiomics correlation analysis linking microbial and metabolic shifts to host phenotypes. (A) Spearman correlation heatmap between key gut microbiota and lipid metabolism markers. (B) Spearman correlation heatmap between differential intestinal metabolites and lipid metabolism markers. *, **, *** indicated statistically significant differences: *P* < 0.05, *P* < 0.01, and *P* < 0.001.

To understand how these microbial shifts translate into physiological benefits, we integrated the metabolomics profile with KEGG pathway enrichment. The analysis revealed that PPP-1 modulates host metabolism through two distinct metabolite-driven pathways:

First, PPP-1 regulates lipid metabolism via the PPAR signaling axis. We observed restored levels of 12-hydroxydodecanoic acid and 9-oxo-octadecadienoic acid. These metabolites are bioactive oxylipins derived from the bacterial metabolism of polyunsaturated fatty acids. Their elevation aligns with the enrichment of “Linoleic acid metabolism” and “α-Linolenic acid metabolism” in our KEGG analysis. Previous studies indicate that gut commensals like *Lactobacillus* and *Bifidobacterium* can metabolize dietary fatty acids into these bioactive intermediates, which function as PPAR agonists.^60^ These observations suggest a direct functional linkage between PPP-1-induced microbial shifts and the modulation of host lipid metabolism through microbiota-derived metabolites and associated signaling pathways. The activation of PPAR promotes hepatic fatty acid oxidation and reduces triglyceride accumulation.^61^ Our correlation analysis supports this mechanism, as these oxylipins were positively correlated with the protective HDL-C and negatively correlated with metabolic risk markers.

Second, PPP-1 alleviates hepatic nitrogen stress via amino acid metabolism. The HFD group showed accumulation of N_2_-succinyl-l-ornithine and D-Octopine, markers that were successfully normalized by PPP-1. Ornithine is a central intermediate in the urea cycle, and the accumulation of its succinylated form suggests a bottleneck in ammonia detoxification.^62^ Similarly, D-Octopine accumulation is indicative of HFD-induced stress and dysregulation of l-arginine pathways.^63^ The normalization of these markers, which corresponds to the KEGG enrichment of “arginine and proline metabolism” implies that PPP-1 restores core hepatic nitrogen balance, potentially by suppressing the Streptococcus-driven dysregulation of these precursors.

Finally, the “ABC transporters” pathway emerged as the most significant hit in the KEGG analysis. ABC transporters are pivotal for the transmembrane efflux of lipids, sterols, and bile acids. The cumulative effect of restoring the gut microbiota and normalizing metabolic signaling likely reinstates the function of these transporters, facilitating the clearance of excess hepatic cholesterol and lipids.^64^ Collectively, these findings demonstrate that PPP-1 mitigates hepatic steatosis not only by directly targeting hepatocytes but also by reshaping the gut environment to promote fatty acid oxidation and relieve metabolic stress. Taken together, these results demonstrate that PPP-1 modulates host metabolism through a coordinated microbiota–metabolite–pathway axis, linking specific bacterial taxa to defined metabolic pathways and physiological outcomes.

While this work establishes PPP-1 as a promising metabolic regulator, further studies are warranted. Although the present study provides a comprehensive preliminary structural characterization of PPP-1, a definitive elucidation of glycosidic linkages and sequence information will require advanced techniques such as NMR spectroscopy and methylation analysis. Due to the limited availability of the purified fraction, high-resolution NMR spectra could not be obtained in the current study. Future studies will focus on large-scale preparation to enable in-depth structural resolution. In addition, the causal role of gut microbiota in mediating the observed metabolic effects should be validated through microbiota transfer experiments. It will also be important to explore potential synergistic interactions between PPP-1 and other bioactive phytochemicals naturally present in pomegranate peel, which may further enhance its biological activity. These efforts will support the development of PPP-1 as a sustainable, high-value functional food ingredient for metabolic health.

In conclusion, this study reports the isolation and characterization of a neutral, glucan-rich polysaccharide, PPP-1, from pomegranate peel and provides the first comprehensive evidence of its potent ability to counteract high-fat diet-induced metabolic disorders. PPP-1 exerts a dual mechanism of action. First, it modulates hepatic lipid metabolism through activation of the SIRT1/AMPK signaling pathway, thereby suppressing the lipogenic transcription factor SREBP-1c and inhibiting de novo lipid synthesis, as demonstrated in both cellular and animal models. Second, PPP-1 functions as a prebiotic that remodels the gut microbial ecosystem, mitigating HFD-induced dysbiosis, reshaping the cecal metabolome, particularly pathways associated with lipid and cholesterol metabolism, and restoring key metabolites linked to metabolic homeostasis. Collectively, these findings elucidate the molecular basis for the lipid-lowering activity of pomegranate peel polysaccharides and underlie the value of pomegranate peel as a sustainable, high-value agro-industrial byproduct with promising applications in functional foods targeting metabolic disorders.

## Data Availability

All data and materials that support the findings of this study are included in the manuscript.
